# Anti-pruritic effect of nemolizumab in hemodialysis patients with uremic pruritus: a phase II, randomized, double-blind, placebo-controlled clinical study

**DOI:** 10.1007/s10157-021-02047-2

**Published:** 2021-03-22

**Authors:** Eriko Kinugasa, Ken Igawa, Hisaki Shimada, Morihiro Kondo, Satoshi Funakoshi, Naoki Imada, Noritomo Itami, Naoki Fukazawa, Ryoko Takubo, Yuichi Kawata, Hiroyuki Murota

**Affiliations:** 1 Showa University Northern Yokohama Hospital, 35-1 Chigasaki-chuo, Tsuzuki-ku, Yokohama, Kanagawa 224-8503 Japan; 2 Dokkyo Medical University, Tochigi, Japan; 3 Shinrakuen Hospital, Niigata, Japan; 4 Rakuwakai Tojiminami Hospital, Kyoto, Japan; 5 Nagasaki Kidney Hospital, Nagasaki, Japan; 6 Nishijin Hospital, Kyoto, Japan; 7 Itami Kidney Clinic, Hokkaido, Japan; 8 Chugai Pharmaceutical Co., Ltd., Tokyo, Japan; 9 Nagasaki University Graduate School of Biomedical Sciences, Nagasaki, Japan

**Keywords:** Hemodialysis, IL-31, Itch, Nemolizumab, Uremic pruritus

## Abstract

**Background:**

The pathophysiology of uremic pruritus (UP), which is characterized by systemic and intractable itching, remains unclear. As interleukin (IL)-31 may be involved, we conducted a phase II, randomized, controlled study to evaluate nemolizumab (anti-IL-31 receptor A antibody) in Japanese hemodialysis patients with UP.

**Methods:**

Patients were randomly assigned (1:1:1:1:1) to one of four double-blind groups (receiving a single subcutaneous injection of nemolizumab 0.125, 0.5, or 2.0 mg/kg, or placebo on Day 1) or an open-label reference group (receiving oral nalfurafine hydrochloride 2.5–5 μg once daily for 12 weeks). The primary endpoint was the difference in the absolute change in pruritus visual analog scale (VAS) at Week 4 between placebo and each nemolizumab group.

**Results:**

The primary efficacy endpoint was not met. The mean change from baseline with all three nemolizumab doses at Week 1, and with 0.5 mg/kg at Week 4, was greater than with placebo. Least square mean differences (95% confidence intervals) in the absolute changes between the placebo arm and each nemolizumab arm were − 2.4 (− 19.7, 14.9) for 0.125 mg/kg, − 8.7 (− 26.6, 9.2) for 0.5 mg/kg, and 0.4 (− 17.0, 17.8) for 2.0 mg/kg. Secondary efficacy parameters including the Shiratori severity score and 5-D itch score failed to show between-group differences. Patients with higher serum IL-31 levels at screening tended to have greater pruritus VAS reductions following nemolizumab treatment.

**Conclusions:**

In this phase II study in patients with UP, the primary efficacy parameter was not met. Nemolizumab was generally well tolerated with no clinically significant safety concerns.

**Clinical trial registration:**

JAPIC: JapicCTI-152961, https://www.clinicaltrials.jp/cti-user/trial/ShowDirect.jsp?japicId=JapicCTI-152961.

**Supplementary Information:**

The online version contains supplementary material available at 10.1007/s10157-021-02047-2.

## Introduction

Uremic pruritus (UP), characterized by systemic and intractable itching, is often without obvious cutaneous symptoms, and occurs in patients with chronic kidney disease [1, 2]. The prevalence of UP is commonly underestimated and many patients with severe symptoms do not receive treatment [3]. A survey conducted in Japan in 2000 revealed that 72.8% of hemodialysis patients had experienced UP and around half of those had suffered from sleep disturbance and reduced quality of life (QoL) [4]. The ongoing Dialysis Outcomes and Practice Patterns Study (DOPPS), conducted in 17 countries including Japan, found that approximately 40% of tracked patients had at least moderate UP; in many of these patients, sleep quality was reduced and UP negatively affected their daily interactions or ability to work [3]. Severe UP has also been associated with a poor prognosis among dialysis patients [5, 6].

There are currently no widely recognized therapeutic guidelines for the treatment of UP, and conventional treatments often prove ineffective [3, 7]. In Japan, nalfurafine hydrochloride (NAL) was launched in 2009; its indication is for ‘improvement of pruritus in dialysis patients (only for cases resistant to conventional treatments)’ [8, 9]. However, the degree of improvement in pruritus in patients receiving NAL has varied considerably [10–12].

Although the exact mechanisms underlying UP still remain uncertain [2], the involvement of interleukin-31 (IL-31) has been suggested [13, 14]. It has been reported that in patients receiving maintenance hemodialysis, the serum IL-31 concentration is higher in patients with pruritus than in those without pruritus [13].

Nemolizumab is a humanized monoclonal antibody targeted against IL-31 receptor A [15, 16], with demonstrated efficacy in reducing the symptoms of pruritus associated with atopic dermatitis [16–19]. The objective of this phase II, randomized, controlled study was to evaluate the efficacy and safety of a single injection of nemolizumab (0.125, 0.5, and 2.0 mg/kg) in Japanese hemodialysis patients with UP.

## Materials and methods

### Patients

The target population was hemodialysis patients with UP whose symptoms were inadequately controlled by standard of care therapy excluding NAL. Key inclusion criteria were age ≥ 20 and < 75 years; hemodialysis (including hemodiafiltration) 3 times weekly for ≥ 12 weeks with a stable regimen before enrollment; a history of inadequate response to systemic therapy (antihistamines or anti-allergic drugs, excluding NAL) or topical therapy, or had received NAL treatment for pruritus within 1 year before informed consent. Patients were also required to have a pruritus visual analog scale (VAS) score [20] of ≥ 20 mm on at least 5 days during the run-in period, and a mean VAS score of ≥ 50 mm. The exclusion criteria are described in the Supplementary Methods.

### Study design and treatment

This phase II, randomized, double-blind, parallel group, placebo-controlled study with an open-label active comparator was conducted at 22 sites in Japan between August 2015 and November 2016 (Supplementary Fig. S1). The study comprised a 4-week screening period, a 1-week run-in period, and a 12-week treatment/observation period. Patients receiving NAL suspended treatment 1 week before the start of the run-in period. Patients were enrolled once the pruritus VAS score reached ≥ 20 mm on at least 5 days and the mean VAS score was ≥ 50 mm; to achieve this, the run-in period could be extended (maximum 8 weeks).

There were five treatment groups in total; patients were randomly assigned to treatment (by the enrollment center) in a 1:1:1:1:1 ratio. Randomization was stratified according to whether the patient was receiving NAL 1 week before the run-in period (yes/no). In the four double-blind groups, patients received a single subcutaneous injection of nemolizumab at doses of 0.125, 0.5, and 2.0 mg/kg, or placebo, on Day 1. In the open-label reference group, NAL was administered orally in the evening at a dose of 2.5–5 μg once daily for 12 weeks, according to symptoms.

The type/dosage of any concomitant agents with an indication for pruritus or moisturizers were to remain stable throughout the study. Rescue therapy after Week 4 (Day 29) was permitted after completion of all observations and tests in patients for whom no pruritus improvement was observed and in patients with reduced efficacy (decrease in pruritus VAS score from baseline < 10 mm) based on the investigator’s judgement.

### Objectives and measures

The primary efficacy objective was to compare the efficacy of nemolizumab and placebo in hemodialysis patients with pruritus. This was measured by the absolute change in pruritus VAS scores from baseline to 4 weeks after the start of administration. The VAS score was recorded daily by patients using a paper questionnaire, to record pruritus intensity in the previous 24 h, on a scale of 0 (no itch) to 10 (worst imaginable itch).

Secondary efficacy objectives were to compare the efficacy of study treatments as assessed by the pruritus VAS score, Shiratori severity score [21], and 5-D itch scale [22, 23] at each evaluation time point. Pruritus VAS was measured as for the primary objective. For the Shiratori severity score, both patients and investigators recorded the extent of daytime symptoms and nighttime symptoms in the previous 24 h based on a pruritus severity scale from 0 (no symptoms) to 4 (severe itch). For the 5-D itch scale, patients evaluated duration, degree, direction, disability, and distribution in relation to their pruritus in the previous 2 weeks using a paper questionnaire; results were converted to a score.

A post hoc analysis was performed to evaluate the proportions of patients achieving a pruritus VAS score of < 30 mm, based on the previously defined cut-off value for mild pruritus [24], and a score of < 10 mm, indicating maximum efficacy. Exploratory analyses were conducted to investigate the following: the impact of treatment on sleep and QoL using patient-reported outcome measures (PROs; the sleep disturbance visual analog scale, the insomnia severity index, and the EuroQoL 5-dimension-5-level questionnaire), the efficacy and safety of nemolizumab using a NAL group for reference, and photographic evaluation of skin symptoms, independently assessed in accordance with Japanese Dermatological Association Atopic Dermatitis Severity Classification (none, mild, moderate, severe, very severe) [25]. A biomarker evaluation was conducted to examine serum IL-31 concentrations at screening and the correlation between serum IL-31 and clinical outcomes following nemolizumab administration. Full details are described in the Supplementary Methods.

The safety of nemolizumab was compared with placebo in hemodialysis patients with UP. AEs were recorded throughout the study duration, regardless of perceived relationship to the study drug, and categorized according to the Medical Dictionary for Regulatory Activities (MedDRA) v18.1. Severity ratings were based on the investigator’s judgement.

### Statistical analysis

It was planned to include 60 patients (12 per treatment group). Based on the absolute change in pruritus VAS scores over 4 weeks in healthy adults and in patients with atopic dermatitis [16], it was assumed that the mean (standard deviation [SD]) difference in the primary efficacy measure between the placebo group and the nemolizumab treatment groups would be 17 (22) mm. Thus, comparison of 12 nemolizumab- and placebo-treated patients per group would yield a statistical power of 71% with a 2-sided level of significance of 20%.

The intent-to-treat (ITT) population included all patients who received ≥ 1 dose of study drug. The per protocol (PP) population excluded patients with major protocol violations or those who received a different treatment from that which they were assigned, patients whose pruritus VAS score was not measured from Week 2 (Day 15) onwards, and patients with a NAL treatment adherence rate of < 70%. The PP population was the main population for efficacy analyses. The safety set included all patients who received ≥ 1 dose of study drug, with patients assessed according to the treatment received.

For the primary analysis, the absolute change in pruritus VAS score from baseline to 4 weeks was compared pairwise between each nemolizumab group and placebo by performing analysis of covariance with the change in scores as the response variable and baseline score as a covariate. The 95% confidence intervals (CI) for the mean difference were calculated. No adjustments were made for multiplicity; missing values were imputed using last observation carried forward (LOCF) methodology. Secondary outcomes were summarized by treatment group at each time point. All statistical analyses were performed using Statistical Analysis Software (SAS) v9.2 (SAS Institute Inc., Cary, NC, USA).

## Results

### Patients

**Fig. 1:**
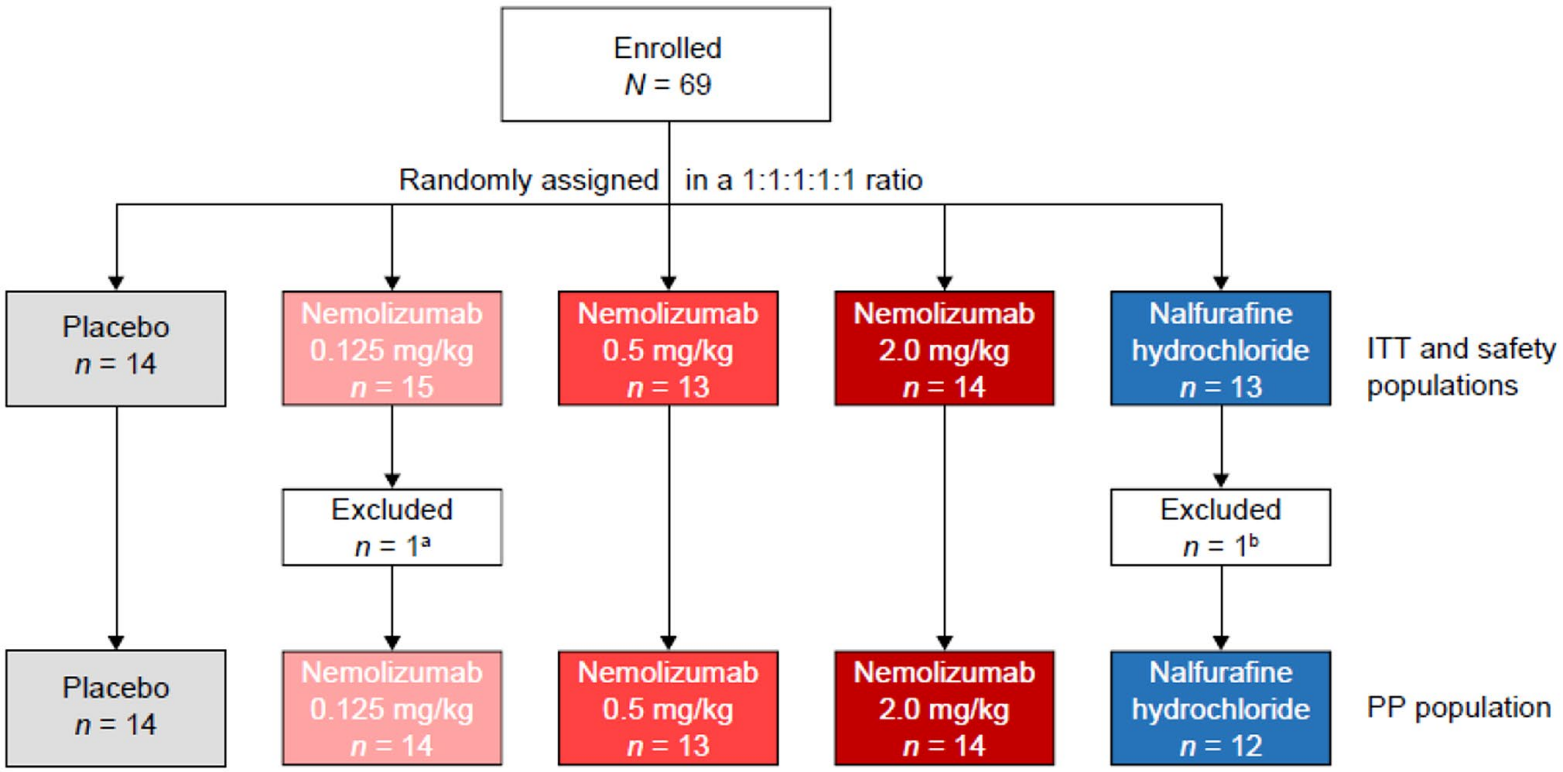
Patient disposition. The ITT and safety populations included all 69 enrolled patients, and the PP population included 67 patients. Overall, 65/69 patients (94.2%) completed the study. One patient in the nemolizumab 0.5 mg/kg group voluntarily withdrew from the study and three patients in the NAL group were withdrawn because of an AE (*n* = 1) or for other reasons (*n* = 2). ^a^Major protocol violation. ^b^Pruritus VAS score was not measured from day 15 onwards. *ITT* intent-to-treat, *PP* per protocol, *VAS* visual analog scale

Patient disposition is shown in Fig. 1. In total, 69 patients were enrolled, comprising 15 in the nemolizumab 0.125 mg/kg group, 13 in the 0.5 mg/kg group, 14 in the 2.0 mg/kg group, 14 in the placebo group, and 13 in the NAL group. All enrolled patients were included in the ITT and safety populations. The PP group comprised 67 patients; one patient in the nemolizumab 0.125 mg/kg group was excluded due to a major protocol deviation (receiving a concomitant prohibited therapy), and one patient in the NAL group was excluded as they had no evaluable pruritus VAS measurement after Day 15.

Baseline demographic and clinical characteristics are shown in Table 1. The mean age, weight and body mass index (BMI) were generally comparable among treatment groups, as were hemodialysis duration, pruritus disease duration, and underlying disease. However, no female patients were included in the nemolizumab 2.0 mg/kg group.

**Table 1:** Baseline demographic and clinical characteristics (per protocol population)

Characteristics	Placebo *n* = 14	Nemolizumab 0.125 mg/kg *n* = 14	Nemolizumab 0.5 mg/kg *n* = 13	Nemolizumab 2.0 mg/kg *n* = 14	NAL *n* = 12
Sex, male	10 (71.4)	10 (71.4)	9 (69.2)	14 (100.0)	10 (83.3)
Age, years	55.1 (11.1)	56.6 (8.4)	58.3 (8.4)	60.1 (9.8)	58.3 (13.0)
Weight, kg	64.4 (20.7)	64.3 (9.5)	64.8 (15.0)	65.8 (15.7)	59.3 (9.6)
BMI, kg/m^2^	23.2 (5.9)	23.9 (3.1)	23.9 (3.9)	24.0 (4.9)	21.9 (2.8)
Pruritus VAS, mm	69.3 (12.4)	67.4 (11.8)	63.6 (7.8)	65.7 (12.3)	69.8 (13.3)
Pruritus disease duration, years	4.2 (4.4)	4.2 (4.1)	6.7 (7.4)	4.9 (4.0)	7.2 (5.6)
Kt/V	1.6 (0.3)	1.5 (0.2)	1.5 (0.3)	1.4 (0.3)	1.7 (0.4)
iPTH, ng/L	196.1 (121.9)	270.2 (288.4)	180.4 (118.2)	247.6 (227.4)	238.0 (238.1)
Calcium corrected, mg/dL	8.9 (0.6)	9.0 (0.5)	9.0 (1.1)	8.8 (0.5)	8.8 (1.1)
Phosphate, mg/dL	5.9 (2.0)	6.1 (1.2)	5.6 (1.2)	6.9 (3.2)	6.3 (0.9)
β2 microglobulin (mg/L)	27.2 (7.7)	28.6 (4.1)	28.8 (6.7)	28.0 (5.7)	28.8 (3.8)
Hemodialysis duration, years	7.9 (5.1)	7.2 (6.1)	7.7 (6.6)	6.0 (4.1)	8.5 (6.4)
History of NAL treatment	3 (21.4)	5 (35.7)	4 (30.8)	3 (21.4)	4 (33.3)
Underlying disease					
Diabetic nephropathy	5 (35.7)	9 (64.3)	5 (38.5)	6 (42.9)	5 (41.7)
Chronic glomerulonephritis	4 (28.6)	1 (7.1)	4 (30.8)	3 (21.4)	4 (33.3)
Nephrosclerosis	3 (21.4)	4 (28.6)	1 (7.7)	1 (7.1)	2 (16.7)
Polycystic kidney disease	0	1 (7.1)	2 (15.4)	2 (14.3)	0

Data are shown as *n* (%) or mean (SD)

*BMI* body mass index, *iPTH* intact parathyroid hormone, *Kt/V* dialysis adequacy (where *K* = dialyzer clearance of urea, *t* = dialysis time, *V* = volume of distribution of urea), *NAL* nalfurafine hydrochloride, *SD* standard deviation, *VAS* visual analog scale

### Efficacy outcomes

**Fig. 2:**
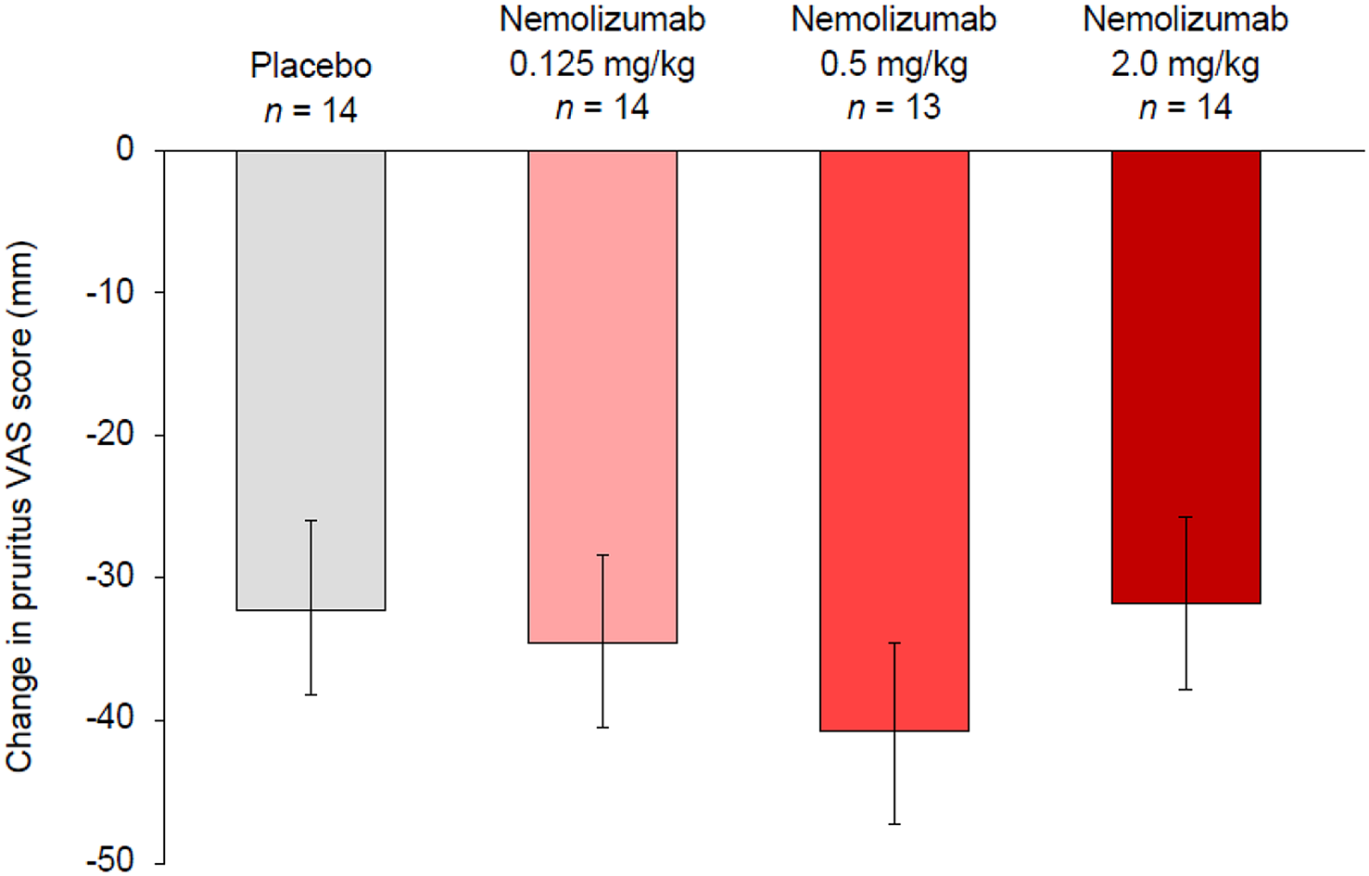
Change in pruritus VAS score from baseline to Week 4 (per protocol population). Data are shown as LSM ± SD. *LSM* least squares mean, *SD* standard deviation, *VAS* visual analog scale

The primary outcome, absolute change from baseline in pruritus VAS at Week 4, is shown in Fig. 2. The least square mean (LSM) absolute changes in pruritus VAS at Week 4 were − 32.1 mm in the placebo group, and − 34.5 mm, − 40.8 mm, and − 31.7 mm in the nemolizumab 0.125, 0.5, and 2.0 mg/kg groups, respectively. The LSM differences (95% CI) in the absolute changes between the placebo arm and each nemolizumab arm were − 2.4 (− 19.7, 14.9) for 0.125 mg/kg, − 8.7 (− 26.6, 9.2) for 0.5 mg/kg, and 0.4 (− 17.0, 17.8) for 2.0 mg/kg nemolizumab. No differences in the data were observed when the results were calculated without using LOCF to impute missing values (data not shown).

**Fig. 3:**
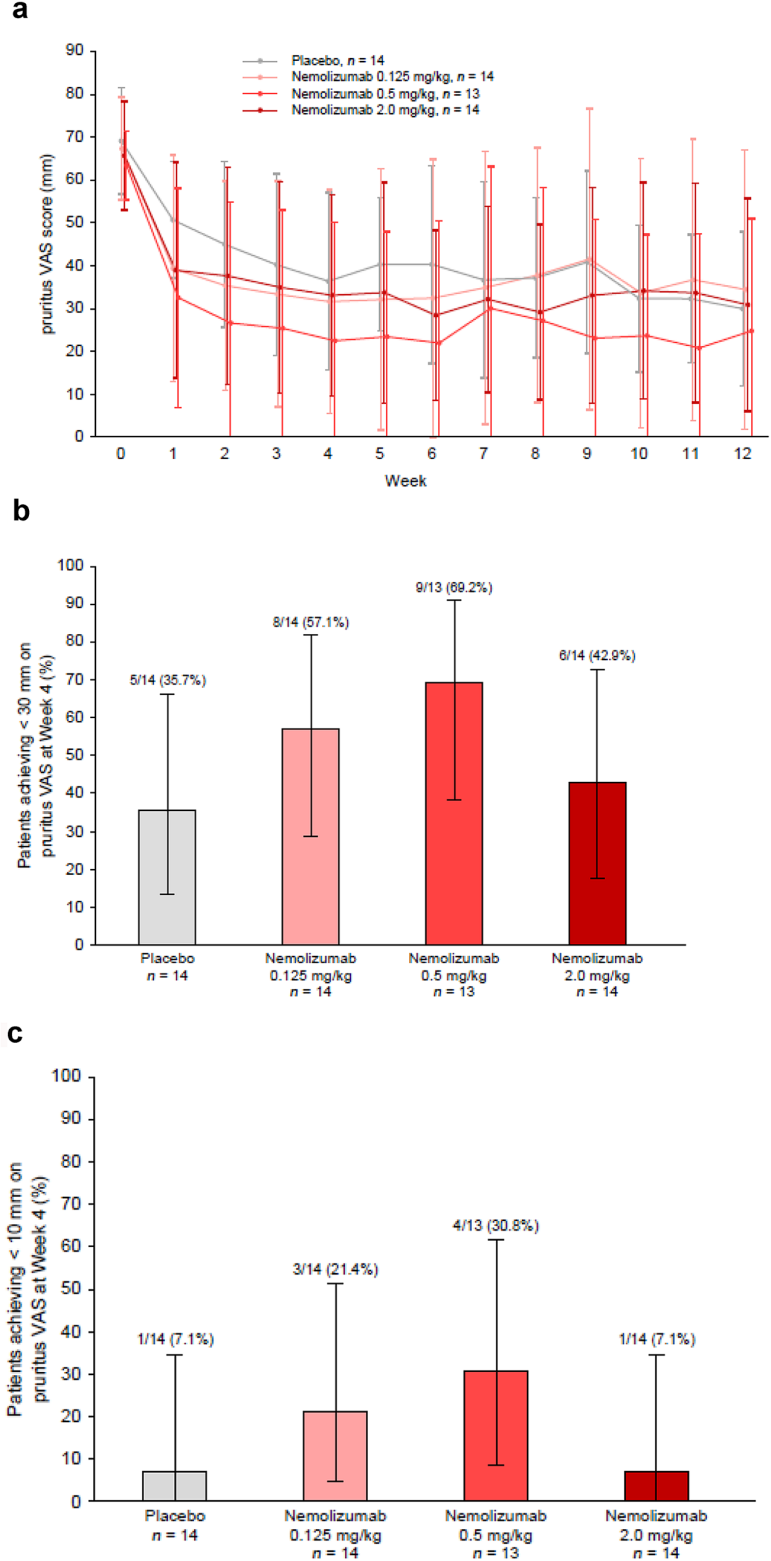
Time course of pruritus VAS^a^ (**a**) and proportion of responders^b^ at Week 4 achieving < 30 mm (**b**) or < 10 mm (**c**) on pruritus VAS (per protocol population). ^a^Data are shown as mean ± SD. ^b^Data are shown as the percentage of patients (95% CI) achieving a score of < 30 mm (panel **b**) or < 10 mm (panel **c**) on the pruritus VAS at Week 4. *CI* confidence interval, *SD* standard deviation, *VAS* visual analog scale

In all nemolizumab groups, improvements in pruritus VAS were observed over time (Fig. 3a). Although no statistical analysis was performed, the mean change from baseline in all three nemolizumab groups at Week 1 (− 27.4 mm, − 30.3 mm, and − 25.9 mm for the 0.125, 0.5, and 2.0 mg/kg doses, respectively) was noticeably greater than in the placebo group (− 18.6 mm). Similarly, the mean change from baseline in the nemolizumab 0.5 mg/kg group at Week 4 (− 40.1 mm) was greater than that in the placebo group (− 32.8 mm). In contrast, the mean changes from baseline in pruritus VAS in the NAL group were − 17.4 mm at Week 1 and − 27.3 mm at Week 4. In the post hoc analysis evaluating the proportion of patients achieving a pruritus VAS score of < 30 mm, it was observed that this proportion at Week 4 in the nemolizumab 0.5 mg/kg group was approximately twofold higher than that in the placebo group (Fig. 3b). The proportion of patients achieving a score of < 10 mm was more than fourfold higher in the nemolizumab 0.5 mg/kg group than in the placebo group (Fig. 3c).

In all groups including the placebo group, improvements in the Shiratori severity score in both daytime and nighttime were observed by Week 4, and there was no clear difference between the placebo group and each nemolizumab group (Supplementary Fig. 2a, b). A similar lack of clear differences between groups was recorded for the 5-D itch scale (Supplementary Fig. 2c).

When the exploratory efficacy endpoints were evaluated, no clear difference between the placebo group and each nemolizumab group in any PRO was observed. In all groups, including the placebo group, improvements in evaluation of skin symptoms using photography were observed by Week 4, and there was no clear difference between the placebo group and each nemolizumab group.

**Fig. 4:**
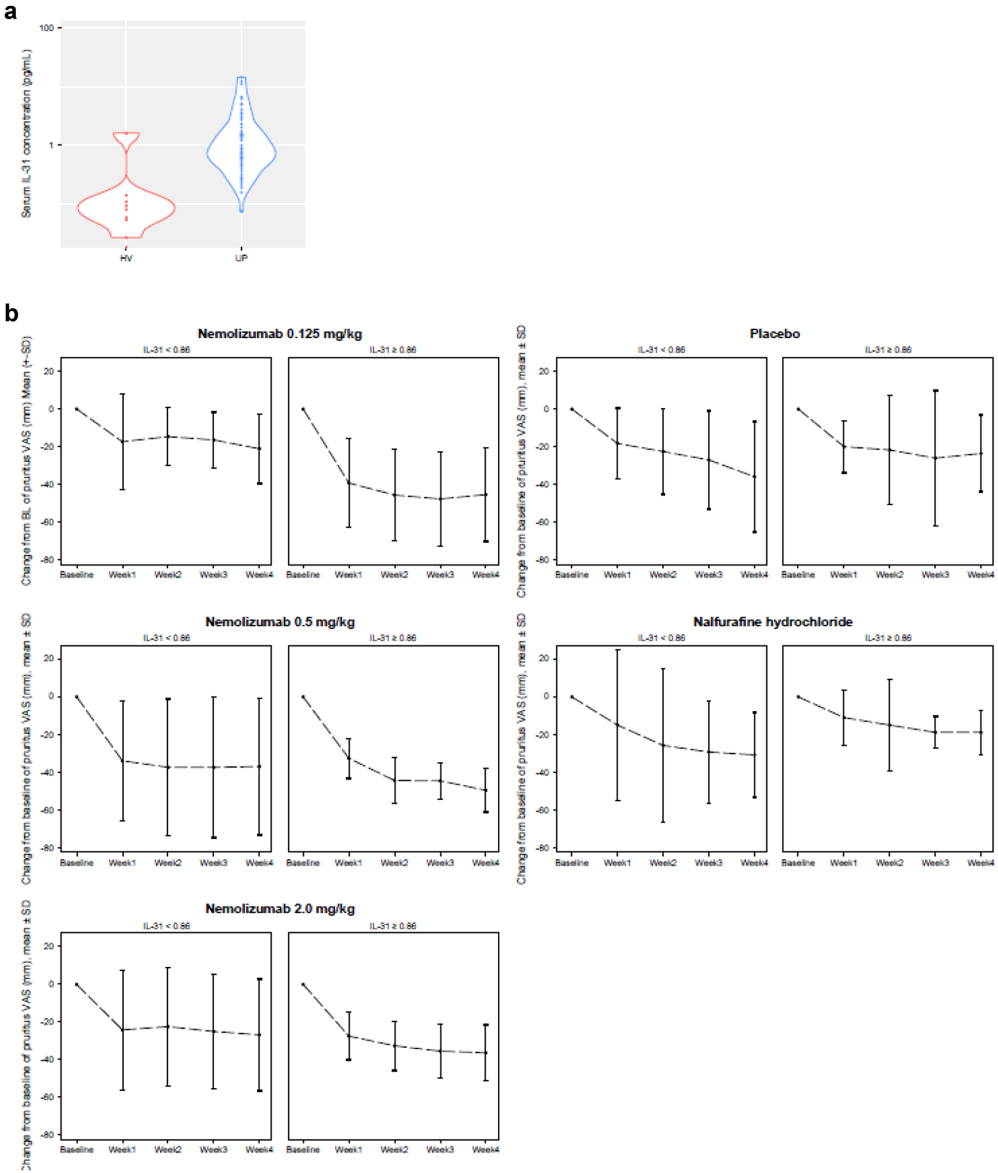
Association between pruritus VAS and serum IL-31 levels. Distribution (log scale) of serum IL-31 concentration in patients with UP and in HV (**a**) and change from baseline in pruritus VAS in patients with UP according to IL-31 category (cutoff: 0.86 pg/mL) (**b**). *HV* healthy volunteers, *IL* interleukin, *SD* standard deviation, *UP* uremic pruritus, *VAS* visual analog scale

Results of the biomarker analysis of the distribution of IL-31 are shown in Fig. 4a; in a post hoc analysis, serum IL-31 levels were higher in patients with UP (*n* = 68) compared with healthy volunteers (HV) (*n* = 20). In the 48 patients with serum samples who received study treatment, there was no correlation between baseline serum IL-31 levels and pruritus VAS values (Supplementary Fig. 3). After treatment, it was observed that nemolizumab-treated patients with IL-31 levels ≥ 0.86 pg/mL showed a reduction in pruritus VAS compared with patients with IL-31 < 0.86 pg/mL (Fig. 4b). This tendency was not observed in either the placebo or NAL groups.

### Safety outcomes

A summary of treatment-emergent AEs (TEAEs) among the 69 patients included in the safety population is shown in Table 2. The incidence rates of TEAEs in the nemolizumab groups (69.2%–73.3%) were similar to the incidence rate in the NAL group (76.9%) and in the placebo group (85.7%). There were no obvious dose-related trends. The most frequent TEAEs were nasopharyngitis, renal anemia, and diarrhea. Treatment-related TEAEs were reported in one patient each in the nemolizumab 0.5 mg/kg (rash erythematous) and 2.0 mg/kg groups (erythema), and in one patient in the NAL group (pruritus and constipation).

**Table 2:** Summary of adverse events (safety analysis population)

Characteristics	Placebo *n* = 14	Nemolizumab 0.125 mg/kg *n* = 15	Nemolizumab 0.5 mg/kg *n* = 13	Nemolizumab 2.0 mg/kg *n* = 14	NAL *n* = 13
Total number of AEs	27	17	18	27	23
Patients with ≥ 1 AE	12 (85.7)	11 (73.3)	9 (69.2)	10 (71.4)	10 (76.9)
Patients with ≥ 1 SAE	0	2 (13.3)	0	1 (7.1)	0
Peripheral arterial occlusive disease	–	1 (6.7)	–	0	–
Pneumonia	–	1 (6.7)	–	0	–
Arteriovenous fistula occlusion	–	0	–	1 (7.1)	–
AEs leading to withdrawal from treatment^a^	–	–	–	–	1 (7.7)^b^
Most frequently reported AEs (≥ 10% in any nemolizumab treatment group)					
Nasopharyngitis	4 (28.6)	4 (26.7)	2 (15.4)	2 (14.3)	2 (15.4)
Renal anemia	2 (14.3)	1 (6.7)	1 (7.7)	2 (14.3)	3 (23.1)
Diarrhea	1 (7.1)	0	3 (23.1)	1 (7.1)	0
Fall	0	0	1 (7.7)	2 (14.3)	0
Excoriation	0	0	2 (15.4)	0	0
Arthralgia	0	0	0	2 (14.3)	0

Data are shown as *n* (%)

^a^Not applicable to the nemolizumab or placebo groups, who received a single dose of treatment

^b^Patient reported constipation and pruritus as the reason for discontinuation

*AE* adverse event, *SAE* serious adverse event

No deaths were reported in any treatment group. Serious adverse events (SAEs) were reported in two patients (13.3%) in the nemolizumab 0.125 mg/kg group and in one patient (7.1%) in the 2.0 mg/kg group (Table 2). None of the SAEs, as assessed by the investigators, were considered to be related to treatment. No patients developed antibodies to nemolizumab after administration.

## Discussion

Uremic pruritus places an additional burden on dialysis patients, and thus well-tolerated therapeutic agents with improved efficacy are needed [3, 6]. Nemolizumab, which targets the pruritogenic cytokine IL-31 [15, 16], has previously shown efficacy against moderate-to-severe pruritus associated with atopic dermatitis [16–19]; thus, this phase II study aimed to evaluate the efficacy and safety of nemolizumab in hemodialysis patients with UP. Differences in the absolute changes in pruritus VAS at Week 4, the primary efficacy outcome, were similar between the placebo group and each nemolizumab group. Likewise, there were no clear differences in the secondary efficacy parameters (pruritus VAS, Shiratori severity score, and 5-D itch scale) at Week 4. However, the mean change from baseline in pruritus VAS with all three nemolizumab doses at Week 1, and in the nemolizumab 0.5 mg/kg group at Week 4, was greater than that with placebo. Furthermore, post hoc analysis showed that approximately twice as many patients in the nemolizumab 0.5 mg/kg group achieved a pruritus VAS score < 30 mm at Week 4 compared with placebo. Overall, no clinically significant safety concerns were identified in the nemolizumab groups.

The placebo response in the double-blind portion of this study was relatively high, and the response to open-label NAL was low. High placebo responses have been reported in other clinical trials on UP [7], and the placebo response is a known confounder in clinical trials conducted in patients with itch [26, 27]. However, a placebo group is necessary, because uremic pruritus may be affected by factors including seasonal climate variations, or environmental changes [28, 29].

In the pivotal phase II NAL study, the mean decrease in pruritus VAS score after 14 days of NAL treatment was 22–23 mm (baseline value 65–69 mm) [9]; although the studies cannot be directly compared, in our analysis, the mean VAS decrease in the nemolizumab groups was 27–36 mm after 14 days of treatment (baseline value 64–67 mm), which is greater than in the NAL study. However, the corresponding decrease in the placebo group in this study (24 mm) was also greater than that reported in the NAL trial (13 mm), making it difficult to compare the relative effectiveness of nemolizumab and NAL between these trials.

Serum IL-31 concentration in patients receiving maintenance hemodialysis has been reported to be higher in those with pruritus than in those without pruritus [13]; this suggests that serum IL-31 concentration in blood may affect or predict efficacy. In a post hoc analysis, serum IL-31 levels in patients with UP were higher than those in HV. Although there was no correlation between serum IL-31 levels at screening and pruritus VAS values at baseline, there was a tendency for patients with higher serum IL-31 levels at screening to have greater pruritus VAS reductions following nemolizumab treatment; this result was not observed in the placebo or NAL groups. These data support the hypothesis that IL-31 may be one cause of UP development.

Our study has some limitations which must be considered. First, the gender imbalance observed in the 2.0 mg/kg arm compared with other treatment groups could have impacted the results in this group. Second, the pruritus VAS has a low detection sensitivity, which may have led to an underestimation of treatment effects. Finally, the high response to placebo, and the low response to NAL compared with historical data, clearly confound the study results; moreover, the different blinding conditions for the study groups further complicates the possible inferences that can be drawn. As such, future study designs will require some ingenuity to improve the outcome detection sensitivity and minimize the placebo effect.

## Conclusions

In this phase II study in patients with UP, the differences in the absolute changes in pruritus VAS at Week 4, the primary efficacy parameter, were not statistically significant between the placebo group and each nemolizumab group. However, the mean change from baseline in all three nemolizumab groups at Week 1, and in the nemolizumab 0.5 mg/kg group at Week 4, was greater than that in the placebo group; moreover, there was a tendency for patients with higher serum IL-31 levels at screening to have greater pruritus VAS reductions following nemolizumab treatment. Nemolizumab was generally well tolerated with no clinically significant safety concerns.

## Supplementary Information

Below is the link to the electronic supplementary material.

Supplementary file1 (DOCX 178 KB)

Supplementary file2 (EPS 856 KB)

Supplementary file3 (EPS 1830 KB)

Supplementary file4 (EPS 2623 KB)
