# Supplementary material for: Anti-pruritic effect of nemolizumab in hemodialysis patients with uremic pruritus: a phase II, randomized, double-blind, placebo-controlled clinical study
Source: Clin Exp Nephrol. 2021 Mar 22;25(8):875–84. doi: 10.1007/s10157-021-02047-2 (PMC8260520; doi:10.1007/s10157-021-02047-2)
Supplement: Supplementary file 1 — Supplementary file1 (DOCX 178 KB) [file 10157_2021_2047_MOESM1_ESM.docx]

**Supplementary Data**

**Supplementary Methods**

***Key study exclusion criteria***

Exclusion criteria included: any active dermatological disease other than UP; the presence of severe cardiovascular disease, or any other clinically significant medical condition; any clinically significant laboratory finding; history of hypersensitivity to an immunoglobulin product; previous treatment with systemic steroids within 4 weeks of enrollment, or NAL within 1 week of enrollment, or initiation or change in dose of any agent to treat UP within 1 week of enrollment, or any investigational product within 120 days before enrollment; previous treatment with nemolizumab; pregnancy, lactation, or unwillingness to use appropriate contraception for 120 days after the start of study treatment; any other condition which made participation unsafe or unsuitable in the opinion of the investigator.

***Biomarker analysis***

Serum IL-31 concentration was measured using an ultra-sensitive enzyme-linked immunosorbent assay (ELISA; Simoa^TM^, Quanterix, Billerica, MA, USA). Samples were obtained from all study patients who consented to the storage of their serum in the clinical repository for future biomarker analysis (*n* = 68), which included 20 patients who subsequently failed screening; data from these 68 samples was used to derive a median cut-off of 0.86 pg/mL at screening. Correlations between IL-31 and clinical outcomes were performed only for patients who received study treatment (*n* = 48), using the IL-31 cut-off value. In a *post hoc* analysis, samples from study patients were compared with commercially available samples from healthy volunteers (HV).

**Supplementary Fig. S1 Study design**


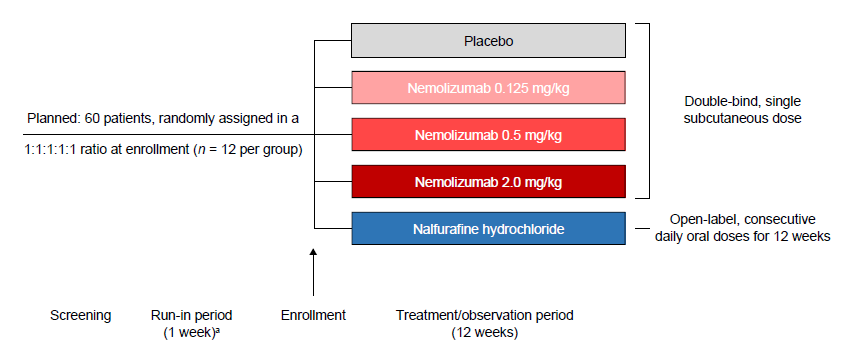


^a^The run-in period could be extended to a maximum of 8 weeks for patients who were receiving nalfurafine hydrochloride up to 1 week before the run-in period.

**Supplementary Fig. S2 Time course of patient-assessed change in the Shiratori Severity Classification** [21] **by treatment group, in the daytime^a^ (a) and at night^b^ (b), and time course of change of the 5-D itch scale^c^ (c) (per protocol population)**


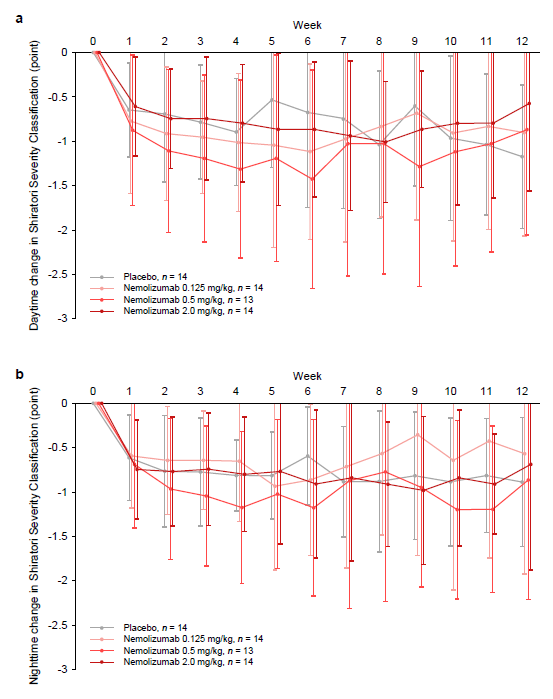


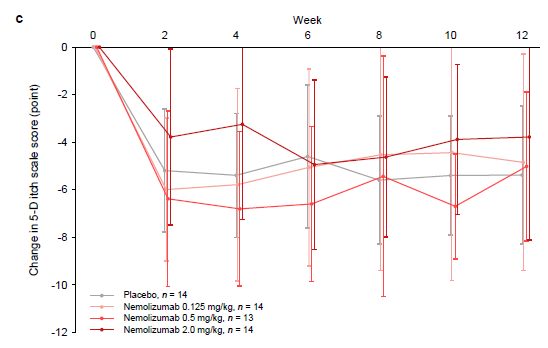


^a^The mean daytime baseline score was 2.3–2.5 points.

^b^The mean nighttime baseline score was 2.0–2.4 points.

^c^Patients evaluated duration, degree, direction, disability, and distribution in relation to their pruritus in the previous 2 weeks. The mean baseline score was 15.0–15.9 points.

Data are derived from patient self-assessment and are shown as mean ± SD.

*SD* standard deviation, *VAS* visual analog scale.

**Supplementary Fig. S3** Serum IL-31 levels at screening and pruritus VAS values at baseline (patients with evaluable serum samples who received study treatment, *n* = 48)


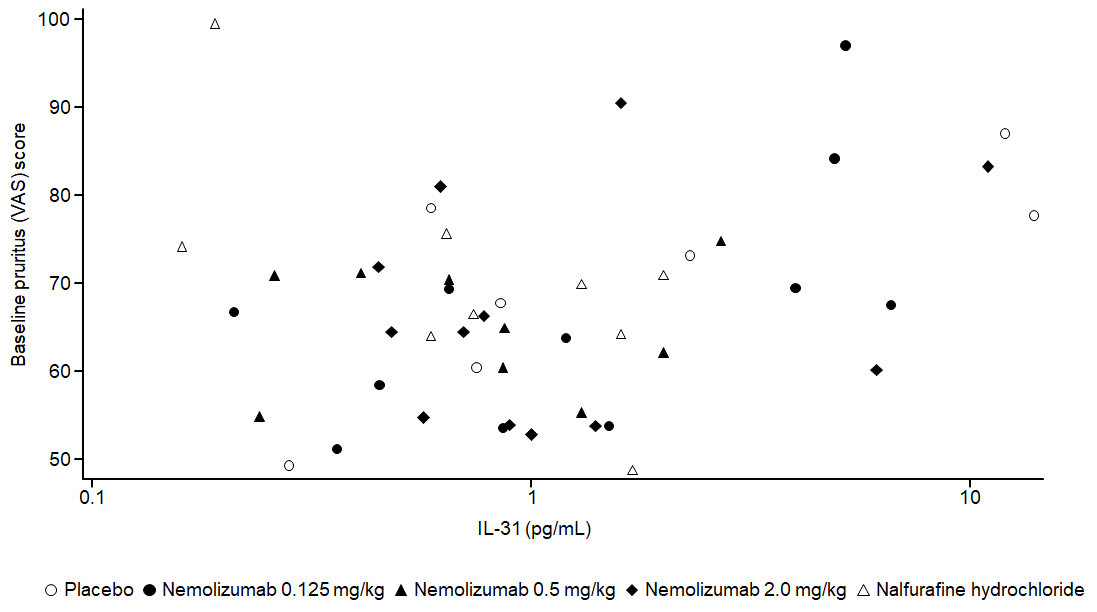


*IL* interleukin, *VAS* visual analog scale.
